# Gender, Mental Health Stigma, and Help-Seeking in Arabic- and Swahili-Speaking Communities in Australia

**DOI:** 10.3390/ijerph21121619

**Published:** 2024-12-03

**Authors:** Shameran Slewa-Younan, Renu Narchal, Ruth Das, Klimentina Krstanoska-Blazeska, Ilse Blignault, Bingqin Li, Nicola Reavley, Andre Renzaho

**Affiliations:** 1 Translational Health Research Institute, School of Medicine, Western Sydney University, Sydney, NSW 2751, Australia; r.narchal@westernsydney.edu.au (R.N.); k.blazeska@westernsydney.edu.au (K.K.-B.); i.blignault@westernsydney.edu.au (I.B.); andre.renzaho@westernsydney.edu.au (A.R.); 2 Centre for Mental Health and Community Wellbeing, Melbourne School of Population and Global Health, University of Melbourne, Melbourne, VIC 3010, Australia; nreavley@unimelb.edu.au; 3 School of Psychology, Western Sydney University, Sydney, NSW 2751, Australia; 4 EMBRACE Multicultural Mental Health Project, Mental Health Australia, Deakin West, WA 2600, Australia; ruth.das@mhaustralia.org; 5 Social Policy Research Centre, University of New South Wales, Sydney, NSW 2033, Australia; bingqin.li@unsw.edu.au

**Keywords:** culturally and linguistically diverse (CaLD), Arabic-speaking, Swahili-speaking, refugees, migrants, Australia, qualitative, mental health, stigma, help-seeking, gender

## Abstract

Australia is an ethnically diverse nation with large numbers of migrants and refugees entering the country yearly. Despite research demonstrating that individuals from culturally and linguistically diverse (CaLD) communities experience an elevated risk of developing a mental illness, mental health services uptake is consistently low. To improve the mental health outcomes of these CaLD individuals in Australia, there is an urgent need to understand barriers to treatment, such as stigma. Research has noted that gender may play a role in mental health stigma and help-seeking. Using a qualitative approach as part of the Embrace Multicultural Mental Health Project, the aim of this study was to explore gender perspectives in mental health stigma and help-seeking among Arabic-speaking and Swahili-speaking individuals in Sydney. A total of five focus group discussions and 18 interviews were undertaken online using Zoom, digitally recorded, transcribed, and thematically analysed. Three major themes were identified. The first theme related to stigma and the fears regarding mental illness being discovered by others. The second theme related to the different approaches to confronting stigma. The last theme related to the various issues considered when identifying sources of help. Our findings suggest that a nuanced approach using the ‘what matters most’ framework can explain how men and women within each community may experience stigma and emphasise different aspects of help-seeking. These findings can help to guide clinical practitioners in delivering gender-specific and culturally sensitive and effective treatment sessions with these CaLD individuals, in addition to offering directions for stigma-reduction initiatives.

## 1. Introduction

Australia is one of the most culturally and linguistically diverse (CaLD) countries in the world, with hundreds of thousands of individuals arriving in the country yearly through various migration streams, including on skilled migrant and humanitarian visas [1]. Between 2014 and 2024, individuals from Iraq, Syria, and Sub-Saharan African countries form the largest group of refugees resettled to Australia [1]. 

### 1.1. Mental Health of CaLD Communities

Migrants, especially refuges and others with refugee-like backgrounds, are considered a priority population due to the stressors they experience, including human rights violation, violence, lack of nutrition, disease, poverty and the resultant physical and mental health impacts [2,3]. Further, indefinite periods of detention and lengthy legal procedures, as well as post-migration experiences such as acculturation challenges due to language barriers, racism, and discrimination all impact mental health [4,5]. Migrants and refugees are at heightened risk of developing mental health disorders due to such experiences [6,7,8,9,10,11].

### 1.2. Help-Seeking Among CaLD Communities

Despite the increased risk of mental health problems, there has been a consistent low uptake of mental health services among CaLD communities in Australia [12,13]. In both Arabic-speaking and Swahili-speaking communities, individuals demonstrate a preference for self-directed and informal sources of help, such as engaging in prayer, talking to friends and family, and consulting religious leaders [14,15,16,17,18]. Additionally, as Islamic tradition values duty of care towards the sick, mental health support is often a family matter in the Arabic-speaking community, with decisions on whether to seek formal support made by the family, particularly men and elders [19,20]. As for the Swahili-speaking population, advice from religious leaders is likely sought because mental illnesses are often conceptualised as spiritual sicknesses, and Western biopsychosocial models are perceived as insufficient to treat these problems [21].

The various barriers postulated to hinder mental health engagement include the high financial cost of mental healthcare, long waitlist for services, low English proficiency of CaLD individuals, a lack of trust in the cultural competency of Australian mental health services, and concerns whether same-language professionals will maintain confidentiality [12,22,23,24,25]. Moreover, individuals report concerns about restrictive treatment such as involuntary hospitalisations or potential problems with visa and immigration renewals following disclosure [26].

Mental health literacy is useful to understand how a range of factors, such as attitudes towards mental health and sources of help, may interact to contribute to patterns of help-seeking [27]. Relatedly, stigma has been consistently identified as a significant barrier to help-seeking [28].

### 1.3. Stigma in CaLD Communities

Goffman (1963) first defined stigma as ‘an attribute that is deeply discrediting’ (p. 3), and ‘describes the situation of an individual who is disqualified from full social acceptance’ (p. 9) [29]. Corrigan and Watson (2002) posited two types of stigmas, namely, public stigma that encapsulates the attitudes an individual perceives his/her society holds toward those with a mental illness, and self-stigma that captures the public’s negative attitudes toward mental illness internalised by the individual with a mental illness [30,31].

Mental health stigma is prevalent in CaLD populations. Among the Arabic-speaking community, stereotypes that mentally ill people are ‘dangerous’ and ‘unpredictable’ have been endorsed across many studies, with the term ‘majnoon’ (madness or insane in Arabic) being a highly stigmatising label indicating ‘deviation from religion’ [32,33,34]. Similarly, research within Sub-Saharan African countries suggested stereotypes that mentally ill people are ‘dangerous to society’, ‘unpredictable’, and ‘hopeless’, with beliefs that mental illness is caused by ‘mapepo’ (demonic/spiritual possession in Swahili) being widespread [12,16,25,35,36].

These negative associations contribute to pervasive denial and rejection of being identified as someone with mental illness and is a strong deterrent to disclosing mental illness or seeking help outside the family [20,22,25]. 

High levels of social distance toward people with mental illness were also endorsed by both communities [32,37]. Compounding the issue of stigma is the possibility of individuals being ostracised following disclosure, which is likely to exacerbate existing mental health problems [12]. Cultural and religious traditions and perspectives have a significant impact on people’s conception and expression of mental disorders [38].

### 1.4. Gender Differences in Stigma and Help-Seeking

Systematic reviews conducted on the general population have concluded that gender is a critical factor in mental health stigma and help-seeking [39,40]. Studies have found that males with mental illness report greater negative attitudes than females, are more likely to be stigmatised, and less likely to disclose mental illnesses or seek professional help [41,42,43,44,45].

Research has suggested that men perceive that the disclosure of difficulties, seeking assistance, and having a mental health diagnosis may lead to being labelled weak, and thus diminishing their masculinity [46,47,48,49,50].

Conversely, a study of help-seeking behaviours in Rwanda found that women were more likely to experience rejection in relationships, leading to divorce and slim chances of remarrying, once mental illness is discovered [51]. Gender roles were found to be a significant consideration when seeking help, as a majority of Rwandan men held the belief that a woman’s most important role is to take care of the home and family, duties which were believed to be negatively affected by mental illness [52]. Shechtman et al.’s (2018) study with Arab adults also found that women reported greater public and self-stigma than men, and that the repercussions of mental illness are greater for women because of their spouses’ reactions [31]. Furthermore, as women represent family honour in Arabic cultural beliefs, greater internalised shame was reported by women with mental illness for ‘betraying the family’s honour’ (p.110) [53].

### 1.5. ‘What Matters Most’ Framework

The ‘what matters most’ framework by Yang et al. (2014) views stigma through interactions between cultural norms, roles, and values in everyday lived activities that impact personhood [54]. This framework was based on previous research by Yang et al. (2007), which hypothesised that participation in everyday social activities that preserves the fundamental values of the group is mandatory for an individual to remain a fully recognised and accepted member of a culture group [55]. Stigma can threaten participation in social activities. Thus, by diminishing a person’s capacity to engage in those everyday social activities, stigma sabotages a person’s membership in that local group, jeopardising his/her personhood [56].

Understanding stigma from the ‘what matters most’ framework helps to overcome the limitation of past approaches that are primarily inductive or focused on general cultural values. For instance, collectivistic values are inherent in both the Arabic-speaking and Swahili-speaking communities where a sense of self is interconnected with the family [17,19,57]. Thus, combining a gender lens and the ‘what matters most’ framework enables researchers to move beyond the assumption that collectivistic values impact the Arabic-speaking and Swahili- speaking communities, and men and women in those communities in the same way, and allows exploration of the specific aspect of stigma that is experienced by that individual in his/her everyday living [56].

### 1.6. Current Study

Given the large Arabic-speaking and Swahili-speaking communities in Australia, an investigation is warranted to understand the possible factors influencing the uptake of mental health services, such as stigma. Additionally, while gender differences were explored in broader populations in Africa and the Middle East, there are no existing studies identifying gender differences within the targeted CaLD populations in Australia. Therefore, this study aims to explore the experiences of mental health stigma by gender among the Arabic-speaking and Swahili-speaking individuals in Australia, and how these factors may impact barriers to and preferences of help-seeking. Using the ‘what matters most’ framework [54], specific cultural and gender nuances will be explored to guide clinical practitioners in delivering culturally sensitive and effective treatment with these CaLD individuals, in addition to offering directions on stigma-reducing initiatives.

## 2. Methods

### 2.1. Study Design and Context

This qualitative research was nested within a larger study led by Western Sydney University that was funded by the Embrace Multicultural Mental Health Project to explore views on mental health help-seeking and stigma among the Arabic-, Swahili-, and Mandarin-speaking communities in Australia. However, only the data pertaining to the Arabic-speaking and Swahili-speaking participants were analysed in this study due to the similarities of these two populations’ migration patterns (refugee-like backgrounds) and premigration experiences.

Ethics approval was sought from Western Sydney University Human Research Ethics Committee (approval number H14608). All researchers had qualifications in health or a health-related discipline. Two bilingual health workers from each of the target communities were employed and trained in the recruitment, screening, and interviewing processes.

### 2.2. Participants

Participants were recruited using the networks and contacts of the relevant investigators and bilingual health workers using both purposive and snowball sampling. Participants were then screened to ensure that they were 18 years of age or older, belonged to the targeted CaLD communities, and had a lived experience of mental illness including those of carers or family members. Participants from the community were involved in a focus group discussion, whereas an individual interview was undertaken only with those identified as a community leader. Leadership included both formal and informal roles, with examples being elders, religious leaders (both Christian and Muslim), or community workers within an ethnic specific and/or trusted organisation. Table 1 summarises the relevant demographic characteristics of participants.

### 2.3. Data Collection

All focus group discussions and interviews were conducted remotely using Zoom due to the COVID-19 outbreak and restrictions in Sydney. Sessions were digitally recorded and transcribed to English by the bilingual health workers.

Prior to the sessions, sociodemographic data of consenting participants were collected in a preliminary phone call by a bilingual health worker, during which participants were asked if they had access to and understood how to operate Zoom.

Each focus group discussion lasted approximately 90 min and was facilitated by two bilingual health workers, whereas the interviews were held with one bilingual health worker lasting approximately 30 min. The focus group discussions and interviews were based on an interview guide developed as part of the larger qualitative research project. Open-ended questions were formulated using a review of literature including direct input from the primary researchers. The questions focused on understanding each community’s conceptualisation of mental illness, including beliefs about aetiology and risk, barriers to seeking treatment such as stigma, and mental health help-seeking preferences. Participants were given an opportunity to review summary content to ensure participants’ perspectives were accurately obtained and interpreted, a verification process that enhanced the credibility of the findings. All community participants received a small (AUD 30.00) electronic supermarket voucher as an appreciation for their participation.

### 2.4. Data Analysis

For this study, a thematic re-analysis of the English transcripts was conducted according to Braun and Clarke’s (2006) data analysis process (see Table 2) [58].

**Table 2:** Braun and Clarke’s (2006) steps of data analysis used in this study.

Step	Description
**Step 1: Data immersion**	English transcripts from the focus group discussions and interviews were read and re-read several times by the primary researcher (analyst) to be familiar with the entire body of data. Informal notes and early impressions were jotted down during this step.
**Step 2: Data coding**	The data were then organised in a meaningful and systematic way using codes. The researcher coded each segment of data according to the domains relevant to the research question, namely, stigma and help-seeking.
**Step 3: Data categorisation**	Fortnightly supervision meetings were held by the researcher with her primary supervisors (S.S-Y. and R.N.) to group initial codes into categories. The connections between the categories were then searched to generate sub-themes and themes. Triangulation of data sources (members and leaders) and collection methods (focus groups discussions and interviews) enabled the researcher to explore a broad range of perspectives and make comparisons within and between the CaLD communities and gender. The regular supervision meetings also enabled the research to consider aspects of analysis including that of reflexivity, also addressed through note keeping.
**Step 4: Theme mapping from categories**	At this step, the preliminary themes were reviewed and modified based on whether the data supported the theme and whether the themes work in the context of the entire data set. The previous papers published under this project were also reviewed to identify if preliminary themes found were in line with previous research and were value-adding to the overall research topic.
**Step 5: Define and finalise themes**	At this step, supervision meetings were held on a weekly basis. The themes underwent a final refinement to identify the ‘essence’ of what each theme is about and whether the sub-themes interacted and related to the main theme. A thematic map (see Figure 1) that illustrated the relationships between the themes was also created to aid in the refinement process. Feedback regarding the cultural sensitivity of the themes and sub-themes found, as well as comments regarding the application of these findings were discussed. This meeting aided in the descriptive validity and transparency of interpretation.
**Step 6: Write up**	Lastly, once the main themes and sub-themes were finalised, the information was written up. This comprised of an existing literature review, reporting of the findings, as well as a discussion of the applications, strengths, and limitations of the findings.

## 3. Results

**Figure 1:**
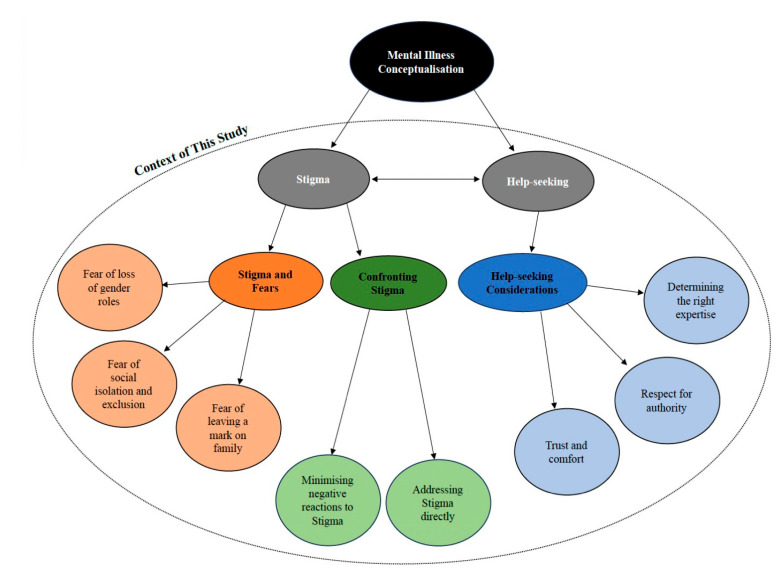
Final thematic map from the study.

Three main themes along with sub-themes were found in this study (see Figure 1). Supplementary Table S1 details the quotes for all the themes and sub-themes.

### 3.1. Theme 1: Stigma and Fear

Stigma was found to be a significant barrier to disclosure, with the fear of specific consequences of stigma driving secrecy behaviours for different groups. This theme comprised three sub-themes.

#### 3.1.1. Sub-Theme: Fear of Loss of Gender Roles

The male Swahili-speaking participants discussed traditional gender roles that men were expected to fulfil, such as being a ‘strong person that can serve my community’ and ‘provide and represent my family’. The presence of a mental illness and the possible associations with weakness were therefore seen to disrupt the man’s ability to fulfil these roles.

In addition, male Swahili-speaking participants shared that the structures and norms in Australia were impacting the changing traditional gender roles because ‘women too can go and work’. As a result, disruptions to identity were further heightened because certain roles that were traditionally fulfilled by men were perceived to be no longer exclusive to them, e.g., ‘man start feeling like they are not that significant in a family’, or maybe ‘you start feeling low self-esteem, they make it seem like sometimes the women don’t need a man’. Therefore, the fear of potentially losing the ability to fulfil traditional gender roles because of mental illness, exacerbated by the changes in the post-migration society, was seen as playing a role in keeping mental illness hidden from others by the Swahili-speaking male participants.

#### 3.1.2. Sub-Theme: Fear of Social Isolation and Exclusion

Across both genders of Swahili-speaking participants, mental illness was kept as a secret, even from friends and family because of the fear of gossiping.

Participants said that ‘if someone is seeing a counsellor, and someone happened just to see the person walking in a counselling service, all the news will be spread in the community’. The consequences they envisioned will be harsh and that ‘it can cause that particular person to feel rejected in the community’ and ‘make the person feel that maybe they are discriminated against’.

Professional confidentiality to protect client privacy in formal mental health services is an ethical requirement. However, due to being part of a small and close-knit community, Swahili-speaking participants still reported the fear of mental health workers or translators gossiping within the community. A community leader shared that some members will not attend a session if it requires an interpreter as, ‘they don’t trust those people, because they are people from their community, and they think they won’t keep confidentiality’.

Therefore, the possibility of isolation and exclusion from the community should the mental illness be known by others was motivating Swahili-speaking participants in this study to engage in secrecy. This was evident even in the context of professional mental health services with females describing the importance of being connected to the community.

#### 3.1.3. Sub-Theme: Fear of Leaving a Mark on Family

Apart from public and self-stigma, a female Arabic-speaking community leader alluded to the strong presence of an affiliate stigma, that is, the extension of stigma to the family members of the person with mental illness. She reported that ‘the mentally ill person’s daughters or sisters will lose the opportunity in getting married, even his relatives will lose a good chance in getting married’. This suggests the tangible rippling effects of stigma beyond the individual and onto family members’ lives, such as marriage, with consequences for females being more significant. Therefore, for Arabic-speaking participants in this study, the fear of stigma creating tangible impacts on family members’ lives acts as a significant deterrent to letting others know about the mental illness.

### 3.2. Theme 2: Confronting Stigma

As a result of various consequences of stigma, different approaches to confronting stigma were described. This theme comprised two sub-themes.

#### 3.2.1. Sub-Theme: Minimising Negative Reactions to Stigma

Female Arabic-speaking participants reported an approach to dealing with stigma to enable the individual experiencing a mental illness ‘feel normal like everyone else’ and ‘don’t think he is mentally ill’. The priority is to ‘deal with him calmly’ and to prevent unpleasant reactions or emotions from the individual, such as anger and shame.

Because of the preference for this indirect approach, a female Arabic-speaking community leader suggested using socially acceptable terminologies whilst running mental health support programs. For instance, she shared that community members did not turn up for the events termed as a session on mental health. However, when she ‘changed the term to mindfulness and relaxation session, they welcomed the idea and attended’.

Therefore, taking on an indirect strategy to minimise negative reactions and ensure greater acceptance and engagement where conversations tackling mental health are phrased within a wellbeing or relaxation context rather than against a backdrop of mental illnesses was preferred.

#### 3.2.2. Sub-Theme: Addressing Stigma Directly

Conversely, both Arabic-speaking and Swahili-speaking male participants reported a more direct approach in addressing mental health-related stigma. They viewed that ‘stigma is one important aspect that you need to break down’ and that people need to be educated about stigma, which will ‘allow the sick individuals to acknowledge and accept that I have a problem and I need to seek for help’.

Because of this direct approach, community leaders in both the Arabic-speaking and Swahili-speaking groups prioritise programs aimed at discrediting any unhealthy associations that individuals hold about people with mental illness and provide further education about different mental health conditions. For example, an interview participant described the African mental health learning cycle, which is a ‘platform where we go and actually try to break this stigma into different points so that our people can actually grab the opportunity of getting to know what mental health is and how can it be treated, so that they can actually accept to receive the treatment that they need’.

Further, community leaders encouraged ‘courageous people who can speak up about their personal experiences in special interviews which can be documented and broadcasted via YouTube or anywhere’. Lastly, there were discussions regarding training community leaders to talk about these issues directly without shying away from potential negative reactions. For instance, ‘providing trainings or sessions to leaders would help them to speak out about the issues freely. In this way, members will be able to seek help from service providers or health professionals’, and that ‘There should be that talking that is going on. And based on that, passing on the information to the community, that’s when access will come’.

Therefore, this approach actively addresses stigma where it is directly discussed by community members and individuals with mental illness are encouraged to share their experiences so that misconceptions can be addressed, and accurate information can be provided, thus prompting people to seek treatment.

### 3.3. Theme 3: Help-Seeking Considerations

While previous papers have identified various help-seeking preferences, the mechanisms guiding individuals in considering certain sources of help over others is examined here according to gender. This theme comprised three sub-themes. 

#### 3.3.1. Sub-Theme: Trust and Comfort

Consistent to both genders, the Arabic-speaking and Swahili-speaking participants discussed that when determining help sources, it was important to know whether a person can be trusted or is one you feel comfortable with.

The Swahili-speaking participants reported that ‘most people do not feel comfortable to go and see people who are strangers’. They indicated that the Swahili-speaking community tend to trust religious elders, such as pastors and church members, because ‘they can speak what they have in their hearts, because they trust the pastors.’ The Arabic-speaking participants reported that people ‘will seek help from someone he knows not from someone he doesn’t know’ and ‘talk to the people you feel comfortable with’. The Arabic-speaking community tend to trust those they have known for a long time, such as family, or people they perceive can keep a secret, such as religious figures.

#### 3.3.2. Sub-Theme: Respect for Authority

The male leaders in both the Arabic-speaking and Swahili-speaking communities noted that individuals tend to turn to those the community respect and have authority. In both groups, these respected individuals with authority were reported to be elders, community leaders, pastors, and other parental figures such as fathers and uncles.

#### 3.3.3. Sub-Theme: Determining the Right Expertise

Though factors such as trust, comfort, and respect for authority are important in determining help-seeking sources, male participants in both groups noted that sources of help must also have the right expertise to address the issue fully. For instance, an Arabic-speaking male reported that people ‘usually seek help from close friend or the parents and family member but does this person they ask help from is able to help?’. Male Arabic-speaking community leaders expressed concern that sometimes solutions provided to the community were not sufficient or helpful because these sources are ‘not specialists’ in the mental health field. A Swahili-speaking male also reported that individuals often turn to people they share a connection with but ‘the problem does not process to a place where you can try and get solved’.

Furthermore, some gender-specific nuances were noted in the way participants defined the right expertise or approach. For instance, female Arabic-speaking participants reported that mental illness can be resolved with increased participation in the community, like going outdoors to a different physical environment, having close communication with family and friends, and adopting prayers to calm oneself down. The male Arabic-speaking and Swahili-speaking participants reported that, ‘mental health is not like having a broken leg or arms where we can identify there is the issue. Not every human being is able to acknowledge or notice it. This is because, it is something that requires maybe to have some kind of education of some sorts’. Because they held the perspective that mental illness could only be effectively resolved with medical support or from professionals who have had proper training, the male participants in this study emphasised that sources of help needed to also have the right expertise.

## 4. Discussion

### 4.1. Theoretical Implications

Previous research has revealed that stigma is highly prevalent in CaLD groups often acting as a barrier to help seeking, influencing sources of help sought [12,14,15,17,25,33,34,35]. This study noted the presence of public, self, and affiliate stigma, which was nuanced by gender, which interacted with previously noted associations within the two target communities to impact disclosure and considerations in help-seeking. For instance, stigma resulted in male Swahili-speaking individuals hiding their mental illnesses due to the fear of losing the ability to fulfil culturally acceptable gender roles. This is consistent with Rugema et al. (2020) and Madsen et al. (2024), who noted that males consider gender roles an important consideration when it comes to mental health-related stigma and help-seeking in CaLD groups [51,59]. This is also consistent more broadly to previous findings that the disclosure of having difficulties, seeking assistance from others, or being diagnosed with a mental health illness can be viewed as impacting notions of masculinity, and therefore playing a role in reduced professional help-seeking [46,47,48,50]. Next, the fear of possible social exclusion in driving secrecy behaviours for the Swahili-speaking participants coincides with previous research that stigma increases the likelihood of experiencing social distance and being ostracized [12,32,37]. The affiliate stigma reported among the Arabic-speaking population revealed in this study is also consistent with findings by Shechtman et al. (2018) and Rugema et al. (2020) suggesting that women experience greater repercussions of mental illness, and that stigma may taint the individual and family members’ identities [19,22,31,51]. The ‘what matters most’ framework helps to explain why these nuances mattered to each group of participants. Firstly, the impacts of post-migration societal norms were important for the male Swahili-speaking participants, as it was suggested that someone who had a mental illness may be viewed as having a diminished ability to engage in the usual activities such as being a bread winer, which was considered necessary for males to remaining a fully recognised and accepted member of their culture group [54]. By diminishing their capacity to engage in those activities, stigma disrupts men’s membership in the Swahili-speaking community and jeopardises their personhood, motivating men to keep mental illness hidden [56]. This may lead to worsening of symptoms, further compromising their ability to fulfil gender roles. Next, considering that the Swahili-speaking community in Australia is a smaller and emerging population, it is not unlikely for professional mental health workers, interpreters, and clients to be known to each other. According to the ‘what matters most’ framework, the possibility of being socially excluded through gossip can be powerful because membership is often vital for individuals to buffer the potential negative impacts of being a minority group in a foreign land [54]. Another interesting finding was the preference for having an indirect approach to addressing stigma reported by female Arabic- speaking participants in this study. It is possible that this was due to the potential reactions of the individual with mental illness and the public, which may spill over onto family members through the effects of affiliate stigma. Apart from the reputational damages and shame of having a mark placed on themselves and their families’ reputation that were discussed in previous studies, avoiding the tangible impacts of stigma onto family members’ lives was perceived as ‘what matters most’ [19,22]. 

### 4.2. Clinical Implications

A core feature of all psychotherapy includes discussion about confidentiality and some form of psychoeducation. This study revealed the significance of addressing confidentiality and the value of addressing assumptions clients may have about confidentiality of service providers, including clinicians and interpreters, particularly in smaller communities such as with Swahili-speaking clients. Additionally, psychoeducation for the Arabic-speaking community might also involve the individual’s entire family unit (where appropriate) such that family members can be informed of the individual’s symptoms, how they might be maintained, and what might be helpful to improve the individual’s presentation. Cognitive Behavioural Therapy (CBT) is the first-line evidence-based treatment for common mental health illness such as depression and anxiet, and the most commonly practiced mode of treatment in Australia [60,61]. This study highlights areas for mental health professionals to adapt their CBT approach when working with Arabic-speaking and Swahili-speaking clients to be culturally sensitive and effective. For instance, behavioural experiments are a common aspect of CBT where the psychologist and client work together to create experiments to test out beliefs and assumptions driving mood symptoms. However, behavioural experiments often require actions that clients engage in a public setting or in the presence of friends and family, which may be problematic for clients with fears related to their community finding out about their mental illness. Consequently, the psychologist may wish to explore other aspects of testing beliefs and assumptions that are within the therapy room without requiring clients to engage in public actions. Depending on ‘what matters most’ to clients, other aspects of CBT can be emphasised instead. For instance, behavioural activation, also another common aspect of CBT, involves helping the client engage in activities that give him/her a sense of mastery or pleasure, which then helps with mood symptoms. Behavioural activation may be well-received in the Arabic-speaking population, as it is more aligned with the notion that having a change in the physical environment, going out into nature, and re-engaging in social activities are effective remedies for mental health problems as described by the Arabic-speaking participants in this study. Moreover, thought-challenging, another key element of the CBT model, may not hold similar salience with CaLD communities. Exploring the beliefs and assumptions that clients have regarding stigma, cultural values, or religion is value-adding if they are reinforcing treatment resistance or significantly impacting the client’s well-being. However, it is important to undertake this exploration in a culturally responsive manner and avoid dismissing or invalidating the client’s cultural background and concerns, which can drive them further away from seeking professional help. It is therefore crucial for mental health professionals to hold ‘what matters most’ to clients, be it religious values or cultural beliefs, and engaging in these factors respectfully while still utilising evidence-based approaches.

### 4.3. Public Health Implications

Findings from this study bear direct relevance to several United Nation Sustainable Development Goals (SDGs) [62]. Specifically, the goals of ‘Gender Equality’ and ‘Reduced Inequalities’ are addressed. This study seeks to understand the unique needs of the males and females within the Arabic-speaking and Swahili-speaking groups in Australia to develop our understanding of their potential help-seeking barriers.

This study also provides some directions for mental health organisations aimed at reducing stigma or improving support for these CaLD communities. For instance, it might be valuable for men’s support groups to be organised to help Swahili-speaking males through the unique experiences of resettlement in Australia. Additionally, it is crucial to organise campaigns providing accurate information about professional confidentiality within formal mental healthcare services for the Swahili-speaking population, as the fear of breach of confidentiality is preventing them from receiving professional help. Turning to the Arabic-speaking population, it was found that females and males have a slightly different approach to addressing stigma. Therefore, recognising preferences in the terms used to describe the stigma reduction programs is important. Moreover, the family unit was described to be the first source of help for various types of problems, but also the main consideration when it comes to potential negative impacts that an individual’s action may have. Therefore, supporting an individual may also require supporting the entire family unit. Lastly, mental health organisations could consider equipping commonly identified sources of help (those who are trustworthy or have authority in the community) with greater mental health support and access to relevant resources and embedding mental health literacy and awareness-building within these commonly identified sources of help. It is vital that these individuals are well-trained and well-equipped to provide effective support. These touch-points might also serve as the most effective channels for important mental health information to be shared directly with those experiencing mental health problems.

### 4.4. Limitations and Strengths of This Study

There are several limitations to note when interpreting the current findings. Firstly, participants, all of whom were living in Sydney, voluntarily participated in the research knowing it was investigating mental health and stigma. Thus, those included in this study could be individuals who hold a more open attitude towards mental illness and a desire to learn more or advocate their views. Additionally, as participants were recruited through the network and contacts of the investigators and bilingual health workers, participants might belong to the same network or know mutual friends belonging to the same network. This potentially enhances the fear of gossiping and could restrict members from openly sharing their views in the focus group discussions for fear that what they shared will be known by others in the community.

Furthermore, there is a possibility of social desirability bias in the results, given the interviews were conducted with community and religious leaders whose views are well regarded in their respective community. Because of their involvement in the community and religious institutions, the perspective offered by leaders may be biased in favour of religious influence on mental health. This was particularly noted when discussing issues related to help-seeking considerations (theme 3). Moreover, while there was an equal number of male and female leaders in the Swahili-speaking interviews, there were significantly more male leaders in the Arabic-speaking interviews. Thus, perspectives offered by the Arabic leaders may be skewed towards a particular gender view. There is also the influence of acculturation and age-related factors on the perspectives reported by participants. Younger participants are more likely to have a faster rate of acculturation and be more open to mental health compared to older participants. Thus, while there were relatively equal numbers of young and older leaders in the Arabic-speaking and Swahili-speaking group, Swahili-speaking community participants were younger. Finally, we did not explore the influence of other factors, such as religion/spirituality or education, on mental health stigma and help-seeking.

Notwithstanding these limitations, the current study had several strengths. Firstly, the investigators and bilingual health workers interacting with participants directly were trained to conduct the focus groups and interviews in a standardised way. They were also equipped to conduct the sessions using the participant’s native languages, helping to build trust with cultural group members and allowing even those with low English proficiency to still voice their views. Additionally, there were frequent meetings among the researchers to help with reflexivity and cultural sensitivity of language used to report findings. Lastly, our study is valuable, as it builds upon the data reported to date by taking a gender-related perspective. Such analyses can help researchers identify if findings of previous studies are reinforced, and if there are new but relevant perspectives to add to the understanding of mental health among the CaLD populations.

## 5. Conclusions

This study provides crucial insights into the way mental health stigma is perceived and experienced by men and women within the Arabic-speaking and Swahili-speaking communities. The impact of changes in traditional gender roles, fear of social isolation, and the rippling effects of stigma onto family members were noted to impact willingness to disclose and the preferred approach to address stigma and considerations when determining help sources. The ‘what matters most’ framework was found to be a useful way of understanding why certain concerns are important to individuals within cultural groups. Future larger-scale research replicating these findings and supplemented with survey methods is required to examine the extent to which these findings apply to target communities across Australia.

## Figures and Tables

**Table 1:** Demographic characteristics of participants in this study.

	Arabic-Speaking		Swahili-Speaking	
	Focus Group Discussions *n* = 3	Interviews	Focus Group Discussions *n* = 2	Interviews
**Gender**				
Male	5	7	8	4
Female	13	3	4	4
**Age**				
18–30	3	-	9	-
30–39	4	-	2	-
40–49	7	2	-	4
>50	4	8	1	4
**Years in Australia**				
1–2	3	-	4	-
3–4	11	-	3	-
>5	4	-	5	-

## Data Availability

The data sets are not publicly available as they contain information that could potentially re-identify individuals, but are available from S.S.-Y. upon reasonable request and with relevant ethical approval.

## Supplementary Materials

The following supporting information can be downloaded at: https://www.mdpi.com/article/10.3390/ijerph21121619/s1, Table S1: Theme and subtheme quotes.
