# Supplementary material for: Gender, Mental Health Stigma, and Help-Seeking in Arabic- and Swahili-Speaking Communities in Australia
Source: Int J Environ Res Public Health. 2024 Dec 3;21(12):1619. doi: 10.3390/ijerph21121619 (PMC11675820; doi:10.3390/ijerph21121619)
Supplement: Supplementary file 1 [file ijerph-21-01619-s001.zip › ijerph-3311711-supplementary.pdf]

Supplementary Table S1: Theme and subtheme quotes

Quotes for Theme 1 – Stigma and Fear	
**Sub Themes**	**Quotes**
**1.1 Fear of loss of gender roles**	*"In African culture, they got these ideas if they found out, mental health is a shame. I cannot be seen in the community as having a mental health illness... I don't look like a strong person that can serve my community, I cannot provide, I cannot represent my family." (Swahili-speaking Community Leader, Male, 44)*<br>*"When you get here in Australia, you have this thing where, I don't think it necessary a bad thing, women too can go and work. They can also go and do these things. This is well and good but then the fact that women can do these stuffs, the role of a men start to get degraded." (Swahili-speaking Male, 21)*<br>*"And then when it comes to be breadwinner men are supposed to be expert in that, it doesn't mean that person is better than the other. It means you are doing well in your role. So, when another person comes in interfere, then it starts to make you feel like you are not an expert in that thing, and when you feel like that you start feeling low self-esteem and... they make it seem like sometimes the women don't need a man." (Swahili-speaking Male, 21)*<br>*"If we were at home, women were really not the breadwinner, so it was very distinct. The roles were very distinct and then when you come here you guys have similar roles and if you have similar roles there's no gaps in between... You guys are all in the same field and when you are in the same field, man start feeling like they are not that significant in a family." (Swahili-speaking Male, 21)*
**1.2 Fear of social isolation and exclusion**	*"It is so hard for you to trust just in case anyone in the Congolese community start telling others how you feel and what you are going through. Because of that, they are going to tell you, you are crazy and start judging you..." (Swahili-speaking Female, 19)*<br>*"Because you will start telling people what I told you which you were not supposed to. You were supposed to keep that as a secret, it is something that is very important and very dangerous and you can't tell just anyone." (Swahili-speaking Female, 19)*<br>*"If someone is seeing a counsellor, and someone happened just to see the person walking in a counselling services, all the news will be spread in the community." (Swahili-speaking Community Leader, Male, 50)*<br>*"Some of them, they won't attend if they know the session need interpreter. And if someone need to interpret for them as I said before, they don't trust those people. And because they are people from their community, and they think they won't keep confidentiality." (Swahili-speaking Community Leader, Female, 44)*
Quotes for Theme 2 – Confronting Stigma	

**2.1 Minimising negative reactions to Stigma**	*"We are giving him the assurance that we are supporting him and in every stage we are with him. We listen to him, take in consideration his opinion and make him feel normal as it is a normal condition and keep the communication." (Arabic-speaking Female, 41)*
**2.2 Addressing Stigma directly**	*"If you're worried about the stigma and someone labels you... that person is sick not you... we must teach people this." (Arabic- speaking Male, 46)*<br>*"Obviously, the stigma is one important aspect that you need to break down so the message could be that there's nothing wrong." (Arabic-speaking Community Leader, 43, Male)*<br>*"I think something that might assist is for people to be educated or informed about mental health issue being the same as physical health. So once the stereotypes or shame is removed or eliminated, it will allow the sick individuals to acknowledge and accept that I have a problem and I need to seek for help." (Swahili- speaking Male, 59)*<br>*"When somebody is educated about something then they will not be ashamed of it. So you are more able to speak out freely. You will be able to seek for help without feeling ashamed." (Swahili-speaking Male, 38)*<br>*"The mentally ill must realise that he needs to seek help." (Arabic-speaking Community Leader, Male, 63)*<br>*"These organisations must intensify their efforts in creating programs to educate people about mental health." (Arabic-speaking Community Leader, Male, 51)*<br>*"There must be ongoing lectures about the topic of mental health and in order for the people not to be scared from the idea that "I am a person with mental illness". (Arabic-speaking Community Leader, Male, 55)*<br>*"If we have courageous people who can speak up about their personal experiences in special interviews which can be documented and broadcasted via YouTube or anywhere, this would encourage people anywhere who are listening to follow their footsteps in seeking help in mental health." (Arabic-speaking Community Leader, Male, 51)*<br>*"What we have started, for example, this group which we call African mental health learning cycle. And this is the platform where we go and actually try to break this stigma into different points so that our people can actually grab the opportunity of getting to know what mental health is and how can it be treated, so that they can actually accept to receive the treatment that they need." (Swahili- speaking Community Leader, Male, 44)*<br>*"By providing trainings or sessions to leaders would help them to speak out about the issues freely. In this way members will be able to*

	seek help from service providers or health professionals." (Swahili-speaking Community Leader, Male, 51)<br>*"So organisation need to make a step to meet the leaders, talk to them, meet the women leaders, meet the youth leaders, meet the pastor. There should be that talking that is going on. And based on that, passing on the information to the community, that's when access will come." (Swahili-speaking Community Leader, Male, 50)*<br>*"We know that the people like to address the mental health issues with the religious leaders. So, we do need training for the religious leaders in the religious corps in mosques and churches." (Arabic-speaking Community Leader, Male, 59)*
**2.3 Fear of leaving a mark on family**	*"The mentally ill person daughters or sisters will lose the opportunity in getting married, even his relatives will lose a good chance in getting married... therefore the family hide the mentally ill person away and keep it as a secret." (Arabic Community Leader, Female, 59)*
Quotes for Theme 3 – Help-seeking Conisderations	
**3.1 Trust and comfort**	*"Most people do not feel comfortable to go and see people who are strangers and start telling them how you feel it not everyone who feels comfortable with that." (Swahili-speaking Female, 18)*<br>*"So for me personally, I would believe pastor because during those times for me. During that time, he was the one that comforted me and gave me the strength I was looking for and it ended up with a good outcome." (Swahili-speaking Female, 27)*<br>*"In Congolese culture they trust pastors than others, they trust pastors because they can tell the truth, they can speak what they have in their hearts, because they trust the pastors and that's why sometimes I know many issues, I play a role of and giving them some help for them." (Swahili-speaking Community Leader, Male, 51)*<br>*"They trust the people in there are religious. The people they meet in the church, they feel more comfortable to open up to them because they trust them." (Swahili-speaking Community Leader, Female, 44)*<br>*"First thing is trust, if we do not trust if I mention it to someone, the news will spread about me as a crazy person and mentally unstable." (Arabic-speaking Female, 41)*<br>*"You should talk to the people you feel comfortable with until you get past the dangerous stage of this illness. Even if you think that there is no definitive cure for this illness, but you will at least partially improve." (Arabic-speaking Male, 35)*<br>*"The person will seek help from someone he knows not from someone he doesn't know." (Arabic-speaking Community Leader, Female, 49)*<br>*"He talks to a trustworthy person from his family before he goes far and approach the psychiatrist." (Arabic-speaking Community Leader, Female, 49)*<br>*"They go to religion figure and the other one is the close person to them they knew for long and trust for sure." (Arabic-speaking Community Leader, Female, 59)*

	"People in general believe that the religious figure will keep the secret and will provide the right advice based on religion knowledge." (Arabic-speaking Community Leader, Female, 59)<br>*"They usually talk to religious person; they have trust in them, and their secret is safe with them." (Arabic-speaking Interview, Community Leader, Male, 56)*
**3.2 Respect for authority**	*"The Arabic speaking look up to leaders of their community they look up to their uncles and aunts and their fathers and so they will listen to them so having elderly people in the community to assist will go a long way." (Arabic-speaking Community Leader, Male, 43)*
**3.3 Determining the right expertise**	*"They usually seek help from close friend or the parents and family member. But does this person they ask help from is able to help?" (Arabic-speaking Community Leader, Male, 68)*<br>*"When you have a problem, the first place you go automatically is to that person that you guys share a connection... In our community because we are very clouded with religion, the problem usually ends there. The problem does not process to a place where you can try and get solved." (Swahili-speaking Male, 21)*<br>*"Sometimes a spiritual guide can help people, but this person can reach a specific point where he cannot continue, so he refers the individual to the specialists." (Arabic-speaking Community Leader, Male, 55)*<br>*"Arabic community in general have trust in the religion figure, therefore, you find them, even ask them mental health problems and psychological issues. This is not right, he is specialised in religion not in mental health….(Arabic-speaking Community Leader, Male, 56)*<br>*"He can take medications, but I do not think that the medications will help him a lot. It could help him temporary to sleep or relax but communication is the main thing." (Arabic-speaking Female, 20)*<br>*"The family are the medicine." (Arabic-speaking Female, 53)*<br>*"Arabic community finds relieve from daily problems by close communication with each other." (Arabic-speaking Community Leader, Female, 71)*<br>*"The person who suffers from any mental health needs to go outdoors and needs to realise the difference in how he was and what he becomes. Always this person needs to do some activities or stroll outdoors." (Arabic-speaking Female, 42)*<br>*"For sure, if a person is restless and anxious, as soon as he performs the prayers, his psyche relaxes and starting to feel better." (Arabic-speaking Female, 28)*<br>*"When it comes to recovery from illness it is not going to happen because of prayer alone we still need medical attention. Prayers will relax your soul and mental health only." (Arabic-speaking Male, 35)*
